# Guild Notes

**Published:** 1913-08-02

**Authors:** 


### *Guild Notes.*

The opening of the Guild offices at 13 Old Steine was a conspicuous success, and every day found numerous enquirers who were anxious and willing to learn what the new movement stood for and what it hoped to do. At the time of going to press the meeting is not yet over, and it is impossible to say what the net result is to the Guild in new members, but we have the satisfaction of knowing that we have sown the seed which will be carried to all parts of the country, and we trust will bear fruit later on when the member has had time and leisure to digest the objects and aims of the Guild.

According to Dr. Wallace Henry, neither the Leicester Union nor the National Medical Guild can claim to be the first medical trade union. In an interesting and instructive speech he reminded the representatives that William III. granted a charter to the Guild of St. Luke in certain parts of Ireland, and that they were given powers to regulate the rates and charges of their members. Possibly the medical men of William III.'s time were not "gentlemen by Act of Parliament."

In the autumn it will be necessary for the National Medical Guild to take a ballot of its members on the subject of incorporating the "political objects" provision of the new Trade Union Act. The Guild proposes to wait until the autumn, as so many of its members are now taking a well-earned rest and do not desire to be worried with matters medical whilst away on a holiday. At present the powers of the Guild do not permit it to use any portion of its funds in the advancement of any political object, and the Council propose to ask the opinion of the members on the subject.

One result of the Brighton meeting was the formation of still another non-panel body, the co-ordinating centre of which is to be London. Dr. E. B. Turner, we believe, is the president, and Dr. Chas. Buttar the chairman. In the multiplicity of counsellors there may be wisdom, but in the multiplicity of organisations we fear there is disaster, and we hope that in the near future they will all come to see that the course mapped out by this body is sufficiently generous to include the non-panel men as well as the panel. Whilst on this subject we may say that the Guild feels a little sore at the attitude of the non-panel doctors towards it, and we should like to remind them that the first action that the Guild fought and won was solely in their interests (we refer to Heard *v.* Pickthorne). That action was a very difficult and expensive one, and the result should have commanded the confidence of the non-panel doctors. One is almost tempted to say, "Ingratitude thy name is non-panel."

